# Task‐specific associations between holistic processing and individual differences in face memory

**DOI:** 10.1111/bjop.70084

**Published:** 2026-05-13

**Authors:** Bryan Qi Zheng Leong, Ahamed Miflah Hussain Ismail, Alejandro J. Estudillo

**Affiliations:** ^1^ School of Psychology Bournemouth University Poole UK; ^2^ School of Psychology University of Nottingham Malaysia Semenyih Malaysia

**Keywords:** composite face effect, exploratory factor analysis, face memory, holistic processing, inversion effect, Navon's effect, part‐whole effect

## Abstract

Whether individual differences in face identification can be predicted by holistic processing, and whether both share a common underlying mechanism, remain open questions. Past studies exploring this association have produced mixed findings, but they have typically examined only a subset of holistic processing measures, focused largely on identification tasks relying heavily on face perception rather than memory, and/or failed to examine whether the relationship between holistic processing and face identification is face‐specific. The present study is the first to examine how all three traditional measures of holistic face processing—the face inversion, part‐whole, and composite face tasks—relate to individual differences in memory‐based face identification, while also exploring whether these relationships are independent of non‐face object identification. We found that face memory was associated with the face inversion and part‐whole effects, but not the composite face effect. Exploratory factor analyses revealed two mechanisms of holistic face processing. The first component was loaded moderately by face memory, inversion, and part‐whole effects, while the second was loaded strongly by the composite face effect. The findings suggest that face recognition is not facilitated by a single, *unitary* holistic processing mechanism and highlight the need to reconsider how holistic processing is conceptualized and measured.

**Practitioner points**

Recognizing faces via our memory is something we do every day—whether greeting a friend, checking ID, or identifying suspects in security footage. But how our brains achieve this complex task has long been debated. Our study challenges the common assumption that there's a single way humans remember and recall faces ‘as a whole’ (what psychologists call holistic face processing). By comparing different measures of holistic processing and memory, we show that face recognition relies on some, but not all forms of holistic processing. Notably, we found that the association between how we recognize faces and how well we recognize them depends on the task utilized. By examining participants from both European and Asian backgrounds, our findings offer a more inclusive and realistic understanding of face recognition. This matters not only for science, but also for designing better tools in security, healthcare (e.g., for people with face blindness), and technology like facial recognition software. In short, if we want to understand how we recognize faces and why some people are better at it than others, we need to rethink the methods we use and the assumptions we have held for decades.

## BACKGROUND

Extensive research supports the view that the human brain represents faces using highly specialized cognitive mechanisms (McKone & Robbins, 2011). Accordingly, studies have shown that humans process human faces differently from other classes of complex visual stimuli (e.g., non‐face objects; Yin, 1969). One of the properties that makes faces ‘special’ is that they are processed and represented as a ‘whole’ rather than as a collection of individual features, termed as *holistic processing* (Maurer et al., 2002; Sergent, 1984). It involves the automatic and seemingly effortless integration of facial features into a unified, gestalt representation, and is thought to underlie successful face recognition (Jacques & Rossion, 2010; McKone & Robbins, 2011; Nakabayashi & Liu, 2014; Piepers & Robbins, 2012; Rossion, 2008, 2013; Tanaka & Farah, 1993). However, measurements of holistic processing of faces remain grounded in specific operational definitions that still lack a clear construct.

Traditionally, holistic processing has been measured with three classic measures: the face inversion task (Rossion, 2008; Yin, 1969), the part‐whole task (Tanaka et al., 2004; Tanaka & Farah, 1993), and the composite task (Rossion, 2013; Young et al., 1987). The face inversion task shows that memory for faces is impaired when they are seen inverted (i.e., upside down) as opposed to upright, in their canonical orientation (Tanaka et al., 2019). This ‘face inversion effect’ (FIE) is particularly stronger for the recognition of faces compared with other non‐face objects (Bruyer, 2011; Rossion & Gauthier, 2002; Yin, 1969). Furthermore, Maurer et al. (2002) have also shown that the FIE is a result of disrupting holistic, rather than featural, processing. In their study, inverting full faces made participants slower and less accurate at recognizing full faces. However, inverting only individual face parts did not impair recognition of those parts (Freire et al., 2000; Leder & Bruce, 1998, 2000; Suzuki & Cavanagh, 1995).

Despite that, Piepers and Robbins (2012) claimed that the FIE does not necessarily constitute a direct measure of holistic processing. In fact, although the FIE is stronger for faces, this effect is not absent for non‐face objects (see Gerlach et al., 2023). In addition, recent evidence suggests that the FIE paradigm does not directly manipulate holistic processing and impacts face processing quantitatively by simply reducing performance (Gerlach & Mogensen, 2024; Tanaka & Simonyi, 2016). In this sense, some authors have argued that while holistic processing is stronger for upright faces, both upright and inverted faces are processed holistically (Gerlach & Mogensen, 2024; Murphy & Cook, 2017; Richler, Mack, et al., 2011). Thus, it is unclear whether FIE has a qualitative or quantitative impact on holistic face processing (Boutet et al., 2021; Gerlach et al., 2023; McKone & Yovel, 2009; Rossion, 2008). In other words, it is unclear if FIE reflects the complete disruption of holistic processing, a reduction in holistic processing, or the disruption of processes other than holistic processing (for discussion, see also Leong et al., 2024; Gerlach, 2025).

More direct evidence for holistic processing during face recognition can be observed from the composite face effect (CFE; Young et al., 1987; Leong et al., 2024; Murphy et al., 2017; Rossion, 2013) and the part‐whole effect (PWE; Estudillo et al., 2022; Leong et al., 2024; Tanaka & Farah, 1993; Tanaka & Simonyi, 2016). The composite task (Hole, 1994; Rossion, 2013; Young et al., 1987) involves the use of face stimuli created by combining complementary top and bottom halves of two different face identities, split at the horizontal meridian. Aligning the top half of one identity with the bottom half of another identity creates the illusion of a new identity, making it hard to attend to one half of the face while ignoring the other. However, misaligning both halves would eliminate such an effect. In other words, the CFE reflects the interference caused by the irrelevant half of the face when participants are required to process the relevant half. Nonetheless, it remains unclear if the CFE reflects face‐specific mechanisms, as the composite effect has also been observed with non‐face objects of expertise (for a detailed discussion, see Murphy et al., 2017).^1^

In the part‐whole task (DeGutis et al., 2013; Estudillo et al., 2022; Leong et al., 2024; Tanaka et al., 2004), participants are required to recognize a set of ‘target’ faces. They are first shown a target face, followed by two ‘test’ faces. In one case, the ‘whole’ condition, participants are asked to identify which of two ‘test’ faces that differ only by one feature (e.g., the nose) matches the face of a learnt target identity. In another case, the ‘part’ condition, they are asked to identify which of two isolated facial features belong to the target identity. The part‐whole task indexes holistic processing as the advantage to recognize a facial feature when it is presented in the context of the studied face. This is because facial features are integrated into a whole, and therefore any new features lead to an illusion of a new identity, making it easier to tell the two faces apart (Tanaka & Farah, 1993). However, when the features are presented in isolation, features must be discriminated at the individual level, which makes identification harder. Furthermore, the PWE has only been observed for faces but not for non‐face objects (e.g., houses), implying that the PWE reflects the involvement of face‐specific mechanisms (Tanaka & Farah, 1993). Interestingly, when participants had to learn face parts instead of whole faces (as in the original part‐whole task), recognition of the learnt parts in the context of whole faces was worse than when parts were shown in isolation (Leder & Carbon, 2005). Consequently, it remains unclear whether the PWE reflects the integration of facial features or the disruption towards the processing of face parts (Tanaka & Simonyi, 2016).

While some studies have shown that holistic processing plays a pivotal role in face identification (Belanova et al., 2021; DeGutis et al., 2013; Leong et al., 2023; Richler, Cheung, et al., 2011; Wang et al., 2012), other studies have found no support for this association (Audette et al., 2025; Konar et al., 2010; Richler et al., 2014; Ventura et al., 2022; Verhallen et al., 2017; Wong et al., 2021). On the one hand, DeGutis et al. (2013) found that holistic processing, as indexed with the PWE and CFE, was positively correlated with face recognition abilities (FRA),^2^ as measured with the Cambridge Face Memory test (CFMT; Duchaine & Nakayama, 2006). On the other hand, Konar et al. (2010) found that the CFE did not correlate with performances in a simultaneous face‐matching task. In addition, a recent study by Audette et al. (2025) showed that the efficiency of processing individual features within a face, but not the efficiency of perceptually integrating individual features (i.e., holistic processing), predicts individual differences in face identification. Adding further to the lack of a relationship between face identification and holistic processing, Verhallen et al. (2017) found that face processing can be explained by a general factor, termed *f*. Interestingly, they found that holistic processing, as measured with the complete CFE, did not correlate with any of the face measures that loaded onto *f* (Verhallen et al., 2017; but see Richler, Cheung, et al., 2011 for evidence of a correlation). These findings further challenge the view that holistic processing is an important aspect of face identification.

Consequently, it is possible that the mixed findings regarding the role of holistic processing on face identification may be explained, at least partially, by the existence of different measures and/or definitions of holistic processing. For example, Audette et al. (2025) did not directly assess holistic processing but instead inferred it through performance efficiency with combined upright facial parts. Additionally, Konar et al. (2010) and Verhallen et al. (2017)'s study only included the CFE as a measure of holistic processing. This is problematic as recent studies (Lee et al., 2022; Leong et al., 2024; Rezlescu et al., 2017) found that the association between these three traditional measures of holistic processing (e.g., FIE, PWE, and CFE) were weak and/or rather absent. A more recent examination with factor analysis by Boutet et al. (2021) also showed that these three traditional measures did not load onto similar components. For instance, the variability in CFE and PWE loaded onto different factors, while the FIE did not load onto any of these factors. They proposed that the PWE was linked with the integration of face parts as well as their configural relations, while the CFE was linked to utilizing a whole face template and/or configural processing (for discussion, see Rossion, 2013; Richler & Gauthier, 2014). Further, Boutet et al. (2021) speculate that the FIE may be linked with inefficient processing of both holistic and featural information, which might explain why it did not load onto any of the factors. In brief, although several paradigms have been developed to measure holistic face processing, these remain grounded in task‐specific operational definitions, and the field still lacks a unified construct of what holistic processing entails (Gauthier, 2020; Richler et al., 2012; Yovel et al., 2014).

If these holistic processing measures are measuring distinct holistic processing mechanisms, as the reviewed evidence suggests, their relationship with face identification should also vary. In line with this, Rezlescu et al. (2017) found that these three traditional measures had varying associations with face identity perception abilities, as measured with the Cambridge Face Perception Test (CFPT; Duchaine et al., 2007). Specifically, only the FIE and the PWE, but not the CFE, showed weak to moderate correlations with the CFPT. This suggests that different measures of holistic processing might contribute differently to the wide range of individual differences seen in face identity perception. Nonetheless, one critical gap in the literature is the lack of research examining how all three holistic measures relate specifically to *face recognition or face memory—*that is, the successful retrieval from memory of a previously learnt face identity. Investigating the specific relationship between different holistic measures and face memory is important both theoretically and empirically, as previous research has shown that face memory is dissociable from face perception in neurotypical individuals (Bruce & Young, 1986; Estudillo & Bindemann, 2014; Fysh et al., 2020; Liu et al., 2021; Stantić et al., 2021; Weigelt et al., 2014; Wilhelm et al., 2010; but see DeGutis et al., 2025) and in individuals with prosopagnosia (Biotti et al., 2017, 2019; Dalrymple et al., 2014; Jackson et al., 2017; Leong et al., 2024, 2025; Ulrich et al., 2017).

This dissociation between perceptual and memory face identification tasks is perhaps unsurprising, as the cognitive demands for each of them are different. Whereas face perception typically involves identity matching between two (or more) simultaneously presented faces, face memory requires encoding and retrieving an identity (Amado et al., 2025; Burton et al., 2010; Estudillo & Bindemann, 2014; Estudillo & Wong, 2024; Fox & Bindemann, 2020; Stantić et al., 2021). It is therefore possible that holistic processing may contribute differently to face perception and face memory. Indeed, some studies have suggested that perception‐based identification tasks, such as the CFPT, the Glasgow Face Matching Test (Burton et al., 2010), and the Kent Face Matching Test (Fysh & Bindemann, 2018), rely less on holistic processing compared with memory‐based identification tasks. This is not only because the former are positively correlated with tasks involving featural processing, such as similarity judgement (Mishra et al., 2021; Rossion & Michel, 2018), figure matching (Burton et al., 2010; Logan et al., 2016; McCaffery et al., 2018; Megreya & Burton, 2006), inverted face matching (Megreya & Burton, 2006), and Navon local processing (McCaffery et al., 2018), but also because disrupting holistic processing has stronger effects in memory‐based than in perception‐based identification tasks (Boutet & Meinhardt‐Injac, 2021; Estudillo & Wong, 2024). In contrast to perception‐based identification tasks, the CFMT requires learning and short‐term retention of unfamiliar faces, as well as assessing identities across lighting changes, a manipulation that engages multiple face‐selective processes and more effectively captures individual differences in face recognition ability (Mishra et al., 2021). These dissociations highlight the importance of examining how different holistic processing measures relate specifically to memory for faces, rather than perception alone.

Moreover, while previous studies have examined associations between face memory and holistic processing, they did not explicitly test (1) whether these associations were face‐specific, and (2) whether they reflected a shared underlying mechanism. For example, neither DeGutis et al. (2013) nor Rezlescu et al. (2017) examined whether their holistic processing measures were also associated with the processing of non‐face stimuli (e.g., objects; see Gerlach & Mogensen, 2024; Boutet et al., 2021). Furthermore, although such correlational evidence is informative, it cannot determine whether the observed associations between different holistic measures and face memory performance reflect a shared underlying mechanism or instead arise from independent sources of variance (Yong & Pearce, 2013). A previous study conducted an exploratory factor analysis (EFA) to explore the structure of different holistic processing measures (Boutet et al., 2021). However, it did not include a measure of individual differences in face identification, so it remains unclear whether holistic processing and individual differences in face recognition share a common underlying structure.

In addition, existing research has largely focused on Western and/or White samples (Boutet et al., 2021; Verhallen et al., 2017), leaving open the question of ecological validity and whether observed relationships generalize across cultures. This issue is particularly relevant given competing claims that holistic face processing differs across cultural groups (Michel et al., 2006; Tanaka et al., 2004), whereas other work suggests that holistic face processing is universal, as demonstrated across all three traditional measures (Crookes et al., 2013; Mondloch et al., 2010; Wong et al., 2021). The extent to which individual differences in face recognition ability (FRA)—which may influence the magnitude of holistic processing—varies across cultures has rarely been discussed. Studying (1) the relationships among all three gold‐standard holistic paradigms and (2) their relationships to face memory across different cultural groups allows us to evaluate not only whether these associations exist but also whether they are consistent across cultures, providing insight into the potential *universality* of holistic processing mechanisms and their relationship to FRA.

### The present study

The current study aims to systematically examine the relationship between holistic processing and memory‐based identification of faces. To address the gaps outlined above, the current study employed the three traditional measures of holistic processing for faces (the inversion, part‐whole, and composite face task) and a measure of holistic processing for non‐faces (Navon's task), along with objective measures of face (i.e., CFMT; Duchaine & Nakayama, 2006) and non‐face (the Cambridge Car Memory test; CCMT; Dennett et al., 2012) recognition abilities. In addition, we indexed a purer measure of face‐specific processing than in previous work. Specifically, we used the regression approach to isolate the memory processes specific to face recognition by regressing out variance reflecting non‐face object memory in the CCMT. Adopting a more generalizable approach, we also included both an East Asian and a White sample in this study.

First, to examine if holistic processing is associated with memory‐based face identification, we correlated performances in holistic measures with the CFMT. Second, to examine if these holistic measures reflect distinct cognitive mechanisms, we correlated the holistic measures with each other. Furthermore, we also ran an EFA to explore whether distinct holistic processing measures and face recognition ability reflect the same mechanism, and if so, whether this mechanism is specific to faces or extends to non‐face objects.

Based on previous research, we expect that the CFMT, FIE, and PWE will load onto the same component. Performances in the CFMT and the PWE have a common reliance on face memory (Tanaka & Simonyi, 2016), and the FIE has often been shown to be associated with the CFMT and the PWE (DeGutis et al., 2013; Rezlescu et al., 2017). In contrast, the standard CFE has been suggested to reflect different cognitive processes, such as perceptual integration (Rossion, 2013) or configural mechanisms (Richler et al., 2012; Richler & Gauthier, 2014; Boutet et al., 2021). More recently, the standard CFE has also been argued to rely on domain‐general selective attention (Fitousi, 2015, 2020; Ventura et al., 2021, 2022) and to be susceptible to attentional manipulations (Weston & Perfect, 2005). Correspondingly, a key component of the global precedence effect (GPE), indexed by the Navon task, is the ability to selectively attend to parts of non‐face stimuli (Gao et al., 2011; McKone et al., 2013; Ventura et al., 2019). Based on these recent accounts, we therefore expect the CFE and the GPE to load onto a second component reflecting domain‐general attentional processes related to holistic processing. Lastly, we expect performances in Navon's task and CCMT to load onto a third component, reflecting domain‐general recognition processes.

## METHODS

### Participants

An a priori power analysis using MorePower 6.0 (Campbell & Thompson, 2012) estimated that a sample size of 84 is required to obtain an effect size of *r* = 0.3 with a statistical power of 80% (*α* = .05), for a Pearson's test of correlation. This effect size was chosen based on previous studies that have examined individual differences in face recognition and holistic processing (e.g., Audette et al., 2025; Leong et al., 2023). Accordingly, this study recruited a total of 202 young adults: 102 British White (84 females) and 100 Malaysian Chinese (70 females). The age range was similar between British Whites (*M* = 21 years, *SD* = 4 years) and Malaysian Chinese (*M* = 22 years, *SD* = 3 years) participants. All participants were recruited through online platforms such as SONA systems (Douglas et al., 2023), social media, and word of mouth. Participants were compensated with either 1.5 credit hours (British Whites) or 15 Malaysian Ringgits (Malaysian Chinese) for their participation. A digital informed consent was obtained prior to participation. All experimental procedures were approved by the Science and Engineering Research Ethics Committee in Malaysia (approval code: BLQZ250920).

### Apparatus

All the British White participants completed their experiments at a lab based at Bournemouth University. Most East Asian participants completed the experiment at a lab based at University of Nottingham Malaysia. The remaining (*N* = 22) completed it online on their own computers, through the testing platform ‘Testable’ (www.testable.org; Rezlescu et al., 2020) while being on a conference call with the experimenter, as data collection from them was affected by COVID‐19 lockdowns imposed by the Malaysian government. To minimize differences in displayed stimuli size across different computer screens used by all participants, participants were required to adjust the length of a yellow line that appeared on their screens to match the width of a debit/credit card they had in possession. This allowed Testable to calculate how many screen pixels (px) mapped on to 1 centimetre (cm) and scale all stimuli based on this conversion. Adobe Photoshop CS6 and Matlab R2019b (Mathworks, Version 9.7.0.1247435) were used to edit stimuli where necessary (refer to Stimuli and Procedure).

### Stimuli and procedure

Each participant was first briefed about the experiment and was informed that they had to complete two different stages: the ‘evaluation’ stage and the ‘experimental’ stage (see also Leong et al., 2024, 2025). The ‘evaluation’ stage, which was always completed first, included the CFMT and the CCMT. This was followed by the “experimental” stage, which included the part‐whole task, the composite task, the face inversion task and the Navon's task. The order of the face holistic measures was counterbalanced across all participants. However, the Navon's task was always completed last as some research has shown that this task could bias subsequent face processing tasks (e.g., Estudillo et al., 2022; Gao et al., 2011; Lewis et al., 2009; Macrae & Lewis, 2002). Accuracy and reaction time (Navon's task only) were measured and recorded. Faces of both races in the holistic tasks were taken from identical databases, thus, the faces all had similar characteristics (e.g., lightning, size, cropping). To avoid *other‐race effects* (Crookes et al., 2013; Estudillo et al., 2020; Michel et al., 2006; Rossion & Michel, 2011), participants were presented with faces congruent with their own race/ethnicity. To do this, two different versions (one with White faces, another with Asian faces) of face processing tasks (e.g., Cambridge Face Memory test—Chinese; CFMT‐Chi, the face inversion, part‐whole, and composite face task) were created. Here, White participants engaged in face processing tasks that presented only White faces, while Asian participants engaged in tasks that presented only Asian faces. The descriptions of stimuli and procedures mentioned from here on are similar for both cultural groups.

#### Evaluation stage

This stage is comprised of the basic evaluation tasks for evaluating participants' recognition abilities for faces and non‐face objects (CCMT). For White participants, we employed the original version of the Cambridge Face Memory test (CFMT, Duchaine & Nakayama, 2006). For Asian participants, we employed the Chinese version (CFMT‐Chi, McKone et al., 2012) of the CFMT.

##### Cambridge Face Memory Test (CFMT/CFMT‐chi)

Both versions of the CFMT have similar stimuli descriptions and task procedures. In these tasks, six unique target face identities and 46 unique distractor face identities (all men) were used. For each identity, there are three face images taken from different viewpoints (1 left 1/3 profile, 1 full‐frontal and 1 right 1/3 profile). All faces were cropped so that no hair, clothing, or facial blemishes were visible. The faces were embedded in the centre of a uniformly black background (195 × 222 pixels (px); 3.9 × 4.44 cm: width × height). The test contained a total of 72 trials from three different stages (e.g., 18 Learning, 30 Novel and 24 Noise). All trials consisted of three faces (one target and two distractors) and participants were required to select which of the three matched a learnt face by pressing the allocated key. All images in the trials were presented in a fixed order. The maximum score on the CFMT is 72. A score below 42 suggests face identification deficits (Bowles et al., 2009; Dalrymple & Palermo, 2016; Estudillo et al., 2020; Leong et al., 2024, 2025).

##### Cambridge Car Memory Test (CCMT)

The CCMT (Dennett et al., 2012) follows an identical format as the CFMT, with the exception that the stimuli were modified computer‐generated images of actual car models (instead of faces), created using 3D Studio Max. To minimize matching based on easily noticeable visual features, all cars are of the same colour, and no identifying badges, logos, or insignias are visible. Car stimuli for the CCMT were sized approximately 465 × 215 px (9.3 × 4.3 cm) (average across cars and viewpoints). Similar to the CFMT, the CCMT also comprises three stages: learning (18 trials), novel (30 trials), and noise (24 trials). The maximum possible score is 72. Any score above 40 denotes normal recognition ability for non‐face objects (Dennett et al., 2012).

#### Experimental stage

##### Face inversion task

Two sets of 30 face identities (15 males) were used for each task (i.e., total of 60 distinct faces). Face images were those of British White and Malaysian Chinese in their early or mid‐20s in neutral expressions. All individuals were photographed in the same range of poses and lighting conditions in the Face Laboratory at the University of Nottingham Malaysia (for full description, see Kho et al., 2024). The external features (i.e., hair, ears) of face stimuli were cropped so that judgements were based on internal facial features only. The faces were also in greyscale and embedded in a 200 × 250 px (4 × 5 cm) black background (see Figure 1).

**FIGURE 1:**
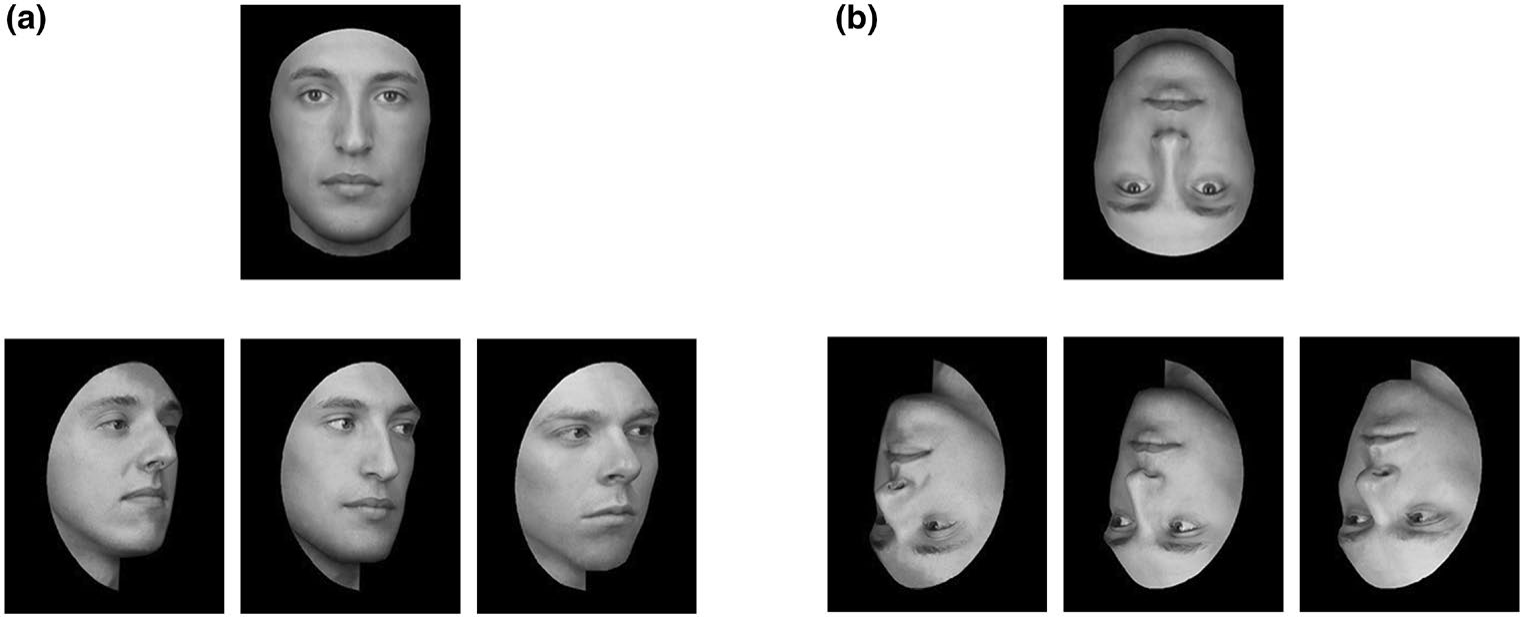
An example of the (White) face stimuli used in the inversion task. A target face (top) followed by three simultaneous test faces (bottom) are shown in each trial: (a) upright and (b) inverted trials.

On any given experimental trial, participants were asked to match one of three test faces (i.e., mid‐profile view) with a target face (i.e., frontal view) in terms of identity. Target identities were also used as test faces (i.e., distractor faces) in trials that have a different target identity. Participants first saw the target face for 400 ms, followed by the three simultaneously presented test images for 2000 ms, and a blank screen until the participant response. Participants were required to press ‘1’ for the face on the left, ‘2’ for the face in the middle, and ‘3’ for the face on the right. The test had a total of 80 trials (40 upright and 40 inverted), presented in random order. Participants were instructed not to tilt their heads when they see inverted faces. Across all trials, each identity was presented twice—once upright and once inverted.

##### Part‐whole task

Face images for this task were taken from Leong et al. (2024), and procedures were similar to those used in previous studies (e.g., Estudillo et al., 2022; Leong et al., 2024; Rezlescu et al., 2017). These images were modified to create new faces with unique combinations of internal features using Photoshop. Target faces were created using either a male or female face template that included the hair and the face outline only. For each (gender) template, six target faces were created by adding internal features such as distinct noses, mouths, and eyes from six different identities (see Figure 2). These six target faces did not share any similar internal features. In total, 12 unique target faces were created (6 males, 6 females).

**FIGURE 2:**
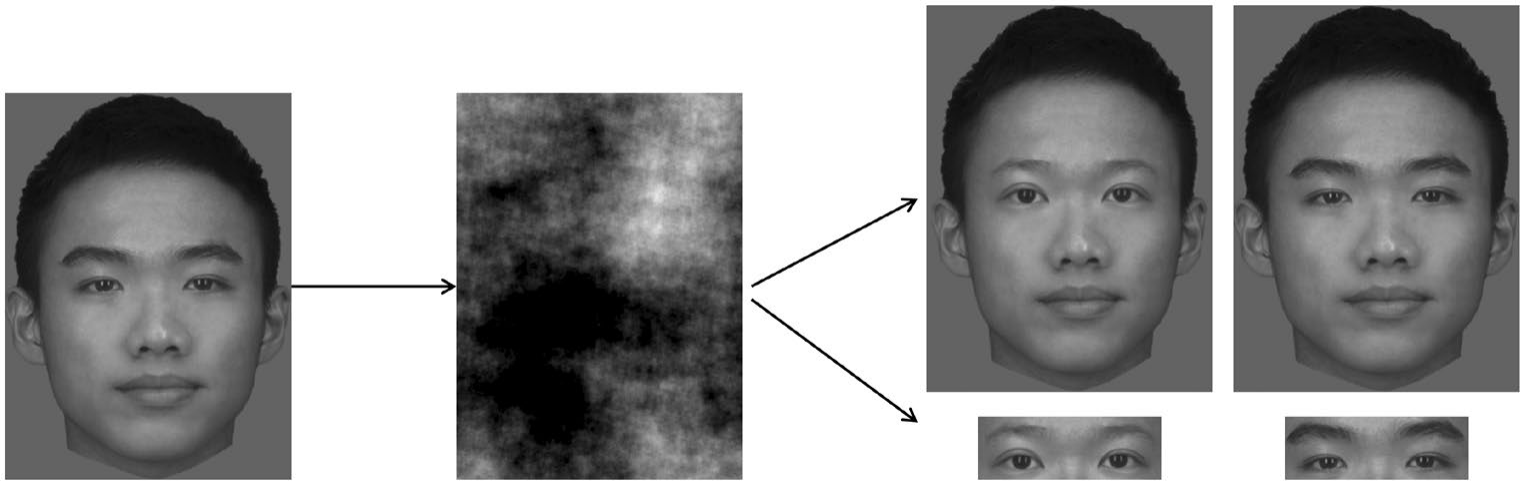
An example of the (Asian) stimuli and procedure used in the part‐whole task. A target face is shown on the left‐hand side, and four test stimuli (in the ‘eyes’ condition) are shown on the right‐hand side: The whole condition (top row) and the part condition (bottom row).

Two types of test stimuli were also created. One of this type of test stimuli consisted of isolated features (mouth, nose, or eyes only) taken from the target faces. The other type comprised of full faces (‘whole foils’) that were created by switching only one of the distinct features of a target face (eyes, nose, or mouth) with that of a different target face. All faces were in greyscale and embedded in a 370 × 500 px (7.4 × 10 cm) grey background. All isolated features were also cropped similarly (e.g., eyes: 234 × 80 px, 4.68 × 1.6 cm; nose: 97 × 77 px, 1.94 × 1.54 cm; mouth: 138 × 71 px, 2.76 × 1.42 cm) from the original face stimuli and the size was kept constant (i.e., same size as the features in full faces) in the experiment.

In each trial, one target image of a whole face was presented for 1000 ms, followed by a phase‐scrambled mask (i.e., Fourier transformed the image and scramble the phase spectrum by multiplying it with a random phase, while maintaining the amplitude spectrum; see Loschky et al., 2007) for 500 ms. The phase‐scrambled mask was created using Matlab R2019b (Mathworks, Version 9.7.0.1247435). In the whole condition, the test images consisted of two full faces: one matching the previously seen target and one foil. In the part condition, the test images were isolated facial features (e.g., two eyes), one from the target and one from a different identity (refer to Figure 2). Participants had to indicate which of the test stimuli matched the target, by pressing one of two allocated keys. There were 144 randomized trials (e.g., 2 conditions × 3 features × 24 trials), with an equal number of male and female targets. Overall, each target face appeared 10 times in total—five times in the whole condition and five times in the part condition.

##### Face composite task

All stimuli were obtained from the Chicago Face Database (Ma et al., 2015). Composite stimuli (for each race) were made from the faces of eight identities (four females). All face images were in greyscale with neutral expressions. Composite faces have their top and bottom halves separated horizontally by a white gap of five pixels. Of the four composites (‘aligned composites’), one of them had a combination of the same identity for the top and bottom halves. The other three were a combination of the top half of one identity with the bottom half of one of the other remaining identities, chosen to match them for gender and face width as closely as possible. These composites were duplicated to create “misaligned composites” where the bottom half of the composite was translated to the right by 25% of its width. These aligned and misaligned composites were used as “target” stimuli.

The bottom halves were always different between the two composites, while the top halves were the same in half of the trials and different in the remaining trials. Participants were asked to ignore the bottom halves and decide whether the top halves of the two composites were the same or different. The participants were required to press ‘Q’ for same and ‘P’ for different. The procedure for this task was adopted from Susilo et al. (2013). The test had 120 randomized trials (40 same‐aligned, 40 same‐misaligned, 20 different‐aligned, 20 different‐misaligned). Each trial began with a fixation cross (1000 ms), followed by two composite faces that were presented sequentially (e.g., the first composite for 200 ms and the second composite for 200 ms) and that was separated by a grey blank screen for 500 ms. The composite faces presented sequentially were either aligned or misaligned composite faces. We used the standard version of the composite task (Leong et al., 2024; Rezlescu et al., 2017).

##### Navon's task

Participants were presented with large letters, either ‘H’ or ‘S’, that were made of either smaller ‘H's or ‘S's. Congruent stimuli had the same alphabetical character for the large and small letters, whereas incongruent stimuli did not (see Leong et al., 2024). The large letters were 278 × 162 px (5.56 × 3.24 cm) in size, and the small letters were 37 × 22 px (0.74 × 0.44 cm) in size. A fixation cross (22 × 22 px; 0.44 × 0.44 cm) was always presented before showing a Navon stimulus. All stimuli were in white and were centred on a 6 × 6 cm black background.

Each participant was presented with four experimental blocks. In two blocks (‘A’), participants were required to report the identity of the global letter (e.g., press the key ‘H’ if the global letter ‘H’ is presented). In the other two blocks (‘B’), they were to report the identity of the local letters. The blocks were always presented in an ABAB order. Participants performed a total of 96 trials across all blocks, where 48 trials consisted of congruent stimuli (e.g., the same identity of local and global letters) and 48 trials consisted of incongruent stimuli. An equal number of stimulus types were presented within each block. Congruent and incongruent trials were randomized within each block. In all blocks, each trial began with a fixation cross presented in the middle of the screen for 1000 ms, followed by the test stimulus shown for 180 ms and a blank screen which remained until a response was recorded. The participants were also required to perform 16 practice trials (equal amount of all four trial types) at the beginning of the experiment.

### Data analysis

Our main measure of recognition abilities for faces and non‐face objects was the scores in the CFMTs and the CCMT. The sum of correct responses in the CFMT/CFMT‐Chi and the CCMT was recorded and analysed. Preliminary analyses were conducted on both sample groups to observe any differences in face and (non‐face) object recognition abilities.

We ran five different types of main analyses. First, to replicate that our different measures of holistic processing perform similar to past studies, we compared the measures of each *condition of interest* (i.e., upright, whole and same‐aligned trials in the inversion, part‐whole and composite tasks, respectively) to their respective *control conditions* (i.e., inverted, part, same‐misaligned trials). Second, we calculated and compared the magnitude of holistic advantage via the regression approach (DeGutis et al., 2013; Leong et al., 2023; Rezlescu et al., 2017), in which the variances of the *control conditions* are regressed from the *condition of interest* to obtain the line of best fit. Using the equation of the line of best fit of the overall scores, each participant's expected score on the condition of interest (i.e., residual scores) was calculated from their performance in the control condition. For the Navon's task, we calculated the global precedence index (i.e., global precedence effect; GPE) for correctly responded trials as the standardized mean difference (Cohen's *d*) between RTs of Local congruent and Global congruent trials. Compared with other Navon indexes, this index offers a purer precedence index as it is not confounded with interference effects (for description, see Gerlach & Krumborg, 2014; Gerlach & Starrfelt, 2018). A higher residual score (for holistic measures) and/or standardized difference (for the Navon's task) represents a stronger holistic advantage.

For the analyses that follow, we used residual scores calculated based on the lines of best fits obtained from each respective participant group (i.e., group residuals), separately. This was done so to ensure that the regression lines were based only on performances within each respective culture, especially since the stimuli within the face tasks conducted were different (i.e., race of faces). Additionally, to obtain a purer measure of face identification that avoids the confound of domain‐general visual identification ability, we computed a residualized face memory score by regressing face memory scores on non‐face object memory scores and retaining the residuals, so that the residuals reflect face identification performance after controlling for non‐face object recognition. Residualized face memory scores were calculated separately for each participant group (i.e., each culture), based on best‐fitting regression models in which face memory scores of each culture (i.e., CFMT for Ws and CFMT‐Chi for EAs) were regressed on their respective CCMT scores.

Third, we correlated the magnitude of holistic advantage (i.e., residual scores for holistic face measures and effect size for Navon's task) against the participant's respective CFMT and residualized CFMT scores, as well as with each other. Fourth, to explore whether the relationship between each of the holistic processing measures and face recognition (both raw CFMT and residualized CFMT) was comparable across cultural groups, we compared the coefficients of correlation (Leong et al., 2023). This was done by transforming the Pearson's correlation coefficient values into Fisher's *z* scores (Hinkle et al., 1988). The analyses were done using the R package *cocor* (Diedenhofen & Musch, 2015).

Lastly, to reveal any factor(s) that emerge from variation in the data and the relationship across the six tasks, we used an EFA with a direct Oblimin rotation, using JASP (v0.19.3; JASP Team, 2025). This was because we found moderate to high correlations within each task (i.e., condition of interests and control conditions), but weak associations between tasks (refer below for analyses; see also Boutet et al., 2021 for detailed discussion on factor analysis involving multiple holistic face measures). Based on the overall sample size, the factor loading threshold of .32 was used (Tabachnick & Fidell, 2007).

## RESULTS

As described by Ramon (2021), reliability of measures should be routinely examined, particularly in holistic face measures (Richler et al., 2014, 2015). Previous studies that used the two evaluation tasks – CFMT and CCMT – have consistently shown that they have high reliabilities, for example, ~.85 and .84, respectively (Bowles et al., 2009; Dennett et al., 2012; Duchaine & Nakayama, 2006; Estudillo et al., 2020; Kho et al., 2024; Murray & Bate, 2020), while the four holistic tasks (face inversion, part‐whole, composite face, and Navon's tasks) has often showed low to moderate reliabilities, for example, ~.2 to ~.7 (DeGutis et al., 2013; Gerlach & Krumborg, 2014; Rezlescu et al., 2017; Richler et al., 2014). To test for internal consistency and/or reliability of our tasks, we calculated Guttman's *λ*_2_ and Cronbach's *α* with the raw scores for each task using the R package *psych* (Revelle, 2023), separated by conditions and sample group. Additionally, using Guttman's λ_2_, we also calculated the reliability of our tasks in computing holistic advantage using the subtraction (for Navon's task) and regression (for face inversion, part‐whole, composite face task) approach (Malgady & Colon‐Malgady, 1991), following the method of calculation in DeGutis et al. (2013) and Leong et al. (2024, 2025). We used Guttman's *λ*_2_ due to its robustness in measuring reliability when dealing with measures that include multiple factors (Callender & Osburn, 1979). In line with previous studies, our four holistic measures showed only modest reliability (e.g., .4 to .7; Gerlach & Krumborg, 2014; Rezlescu et al., 2017), while the evaluation tasks showed high reliability (~.8; see Table 1).

**TABLE 1:** Reliability scores of White (Ws) and Asian (EAs) participants.

Tasks/conditions	Ws		EAs	
	Guttman's *λ*_2_	Cronbach's *α*	Guttman's *λ*_2_	Cronbach's *α*
CFMT	.891	.895	.894	.891
CCMT	.820	.798	.766	.754
CFMT residual	.890	–	.876	–
Inversion				
Upright	.723	.723	.724	.704
Inverted	.677	.561	.409	.347
FIE residual	.563	–	.674	–
Part‐whole				
Whole	.779	.779	.783	.758
Part	.715	.659	.613	.572
PWE residual	.416	–	.681	–
Composite				
Same‐aligned	.834	.834	.866	.868
**Same‐misaligned**	.820	.820	.873	.834
CFE residual	.442	–	.663	–
Navon				
Global congruent	.445	.402	.701	.679
Local congruent	.569	.510	.756	.760
GPE subtraction	.469	–	.607	–

Abbreviations: CCMT, Cambridge Car Memory Test; CFE, composite face effect; CFMT, Cambridge face memory test; FIE, face inversion effect; GPE, global precedence effect; PWE, part‐whole effect.

The maximum achievable score (e.g., sum of correct responses) for the CFMT and CFMT‐Chi is 72, in which our current White participants (Ws) had a mean score of 60.46 (*SD* = 8.33) and East Asian participants (EAs) had a mean of 60.84 (*SD* = 8.26), respectively. Initial independent *t*‐tests revealed that the FRA of EAs and Ws were comparable, *t*(200) = .325, *p* = .746, *d* = −.046 (see Figure 3). These numbers are also similar to reported in previous studies (Estudillo et al., 2020; Estudillo & Wong, 2021; Leong et al., 2023; McKone et al., 2012, 2017). However, our analyses revealed that EAs had an overall higher non‐face object recognition ability (as measured by the CCMT) compared with Ws, *t*(200) = 2.728, *p* = .007, *d* = −.384. Further analysis showed that EAs took significantly longer in the CCMT than Ws (*t*(200) = −11.113, *p* < .001, *d* = −1.564), which could potentially account for the slightly higher performance observed in EAs for the CCMT. Furthermore, multiple Levene's tests confirmed that, across the six measures used, the variance in performance of Ws did not differ from that of EAs (*p* > .05), thus allowing us to proceed with parametric tests (see Figure 3).

**FIGURE 3:**
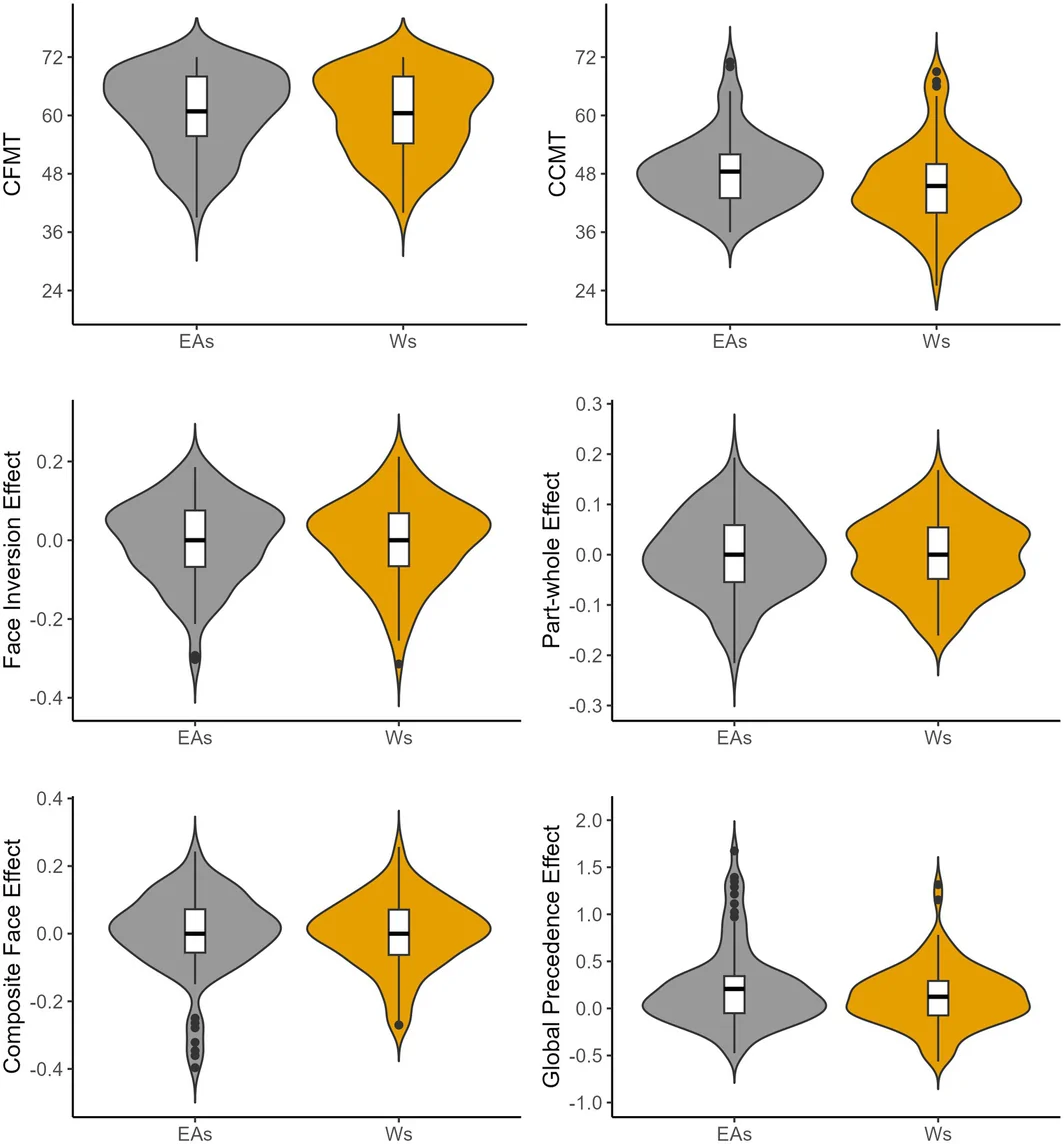
Independent sample *t*‐tests of the six measures between EAs (grey) and Ws (orange). The violin plot represents the density distribution of performance in each group. The horizontal line within the boxplot represents the mean scores, while the top and bottom hinge of the boxplot represent the first and third quartiles. The vertical black line outside of each boxplot represents the 95% confidence interval. Black‐filled circles represent the accuracy scores of individual participants that are outside the 95% confidence interval.

### Face inversion task

In general, participants were more accurate with upright (*M* = .786, *SD* = .113) compared with inverted (*M* = .536, *SD* = .104) trials, *t*(201) = 29.792, *p* < .001, *d* = 2.096, regardless of cultural groups (i.e., pooling data from both groups). This pattern replicates the classic face inversion effect commonly reported in previous studies (e.g., Leong et al., 2024; Rossion, 2008; Yin, 1969). The group comparison analyses revealed a comparable inversion effect (residuals) in EAs (*M* = .008, *SD* = .102) and Ws (*M =* −.008, *SD =* 0.116), *t*(200) = 1.055, *p* = .293, *d* = .148 (see Figure 3). The overall magnitude of the inversion effect, including both EAs and Ws, was associated with CFMT scores, *r*(200) = .385, *p* < .001 (refer to Figure 4). This remained true with the residualized CFMT scores as well, *r*(200) = .372, *p* < .001. When we explored this relationship separately for each participant group, the magnitude of the inversion effect was positively correlated with raw CFMT scores in both Ws (*r*(100) = .314, *p* = .001) and EAs (*r*(98) = .461, *p* < .001) (see Figure 4). The correlation coefficients of both groups were comparable (*z* = −1.215, *p* = .224). The same result was observed with residualized CFMT scores in Ws (*r*(100) = .309, *p* = .002) and EAs (*r*(98) = .441, *p* < .001), and the correlation coefficients were comparable between participant groups (*z* = −1.078, *p* = .280).

**FIGURE 4:**
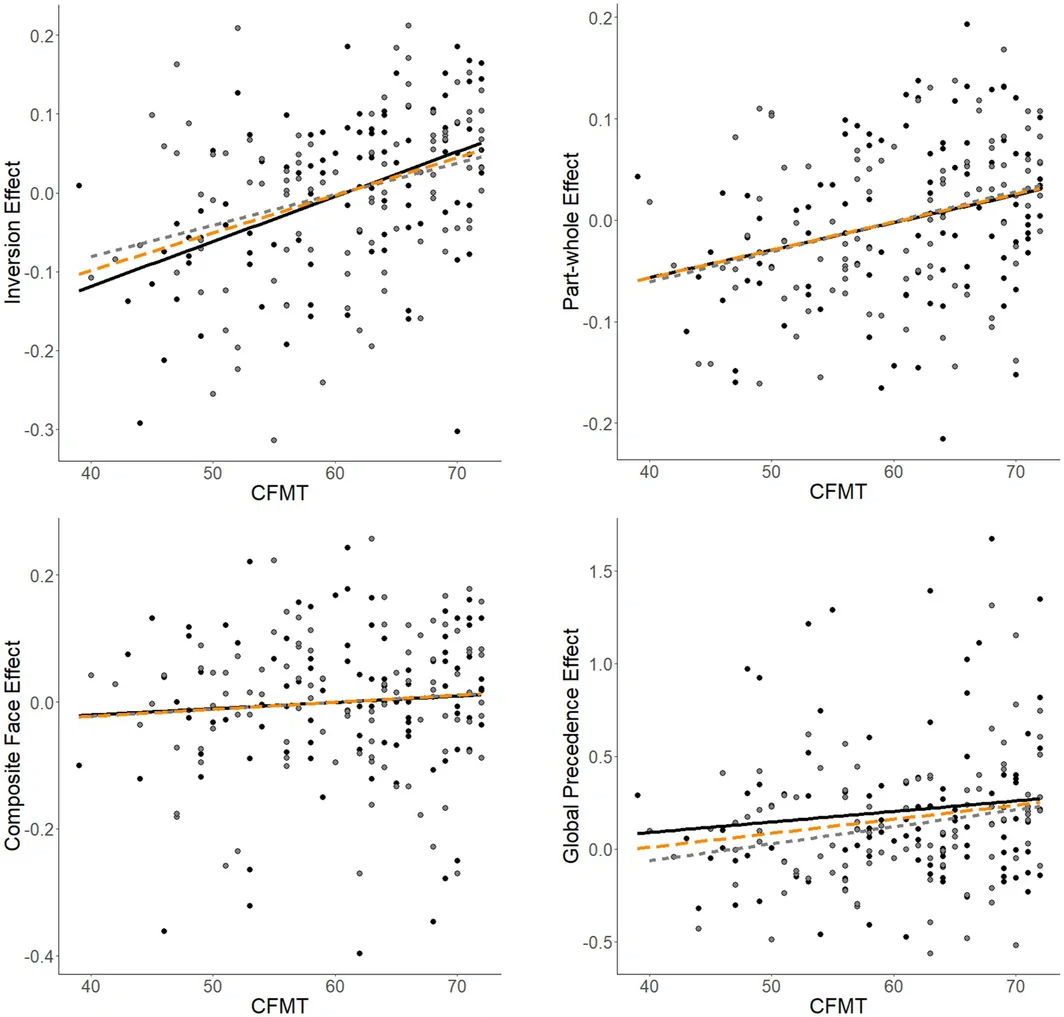
Correlation plots of holistic advantage and face recognition abilities of all participants (orange), Ws (grey) and EAs (black). The grey and black dots represent individual residuals of White (Ws) and Asian (EAs) participants, respectively. The orange dashed, grey dashed, and black solid lines are least‐squares regression fits to individual data of all participants, Ws, and EAs, respectively.

### Part‐whole task

Irrespective of cultural groups, participants performed better with whole (*M* = .777, *SD* = .096) compared with part (*M* = .676, *SD* = .085) trials, *t*(201) = 17.393, *p* < .001, *d* = 1.224. This pattern also replicates the classic part‐whole effect reported in previous studies (e.g., DeGutis et al., 2013; Estudillo et al., 2022; Leong et al., 2024). The group comparison analyses revealed a comparable part‐whole effect (residuals) in EAs (*M* = −.005, *SD* = .080) and Ws (*M =* .005, *SD =* .074), *t*(200) = −.923, *p* = .357, *d* = −.130 (see Figure 3). The overall magnitude of the part‐whole effect was associated with CFMT scores, *r*(200) = .307, *p* < .001 (refer to Figure 4). This remained true when correlated against residualized CFMT scores as well, *r*(200) = .315, *p* < .001. When we explored this relationship separately for each participant group, the magnitude of the part‐whole effect was positively correlated with CFMT in both Ws (*r*(100) = .335, *p* < .001) and EAs (*r*(98) = .281, *p* = .005) (see Figure 4). The correlation coefficients of the part‐whole effect and CFMT were comparable between both groups (*z* = .418, *p* = .676). The same result was observed with residualized CFMT scores in Ws (*r*(100) = .323, *p* < .001) and EAs (*r*(98) = .308, *p* = .002), and the correlation coefficients were comparable between participant groups (*z* = .117, *p* = .908).

### Composite face task

Participants performed better with same‐misaligned (*M* = .810, *SD* = .141) compared with same‐aligned (*M* = .742, *SD* = .154) trials, *t*(201) = 8.232, *p* < .001, *d* = .579, replicating the classic composite face effects reported in the literature (e.g., Leong et al., 2024; Rossion, 2013; Young et al., 1987). The group comparison analyses revealed a comparable composite effect (residuals) in EAs (*M* = −.010, *SD* = .121) and Ws (*M =* .009, *SD =* 0.105), *t*(200) = −1.206, *p* = .229, *d* = −.170 (see Figure 3). The overall magnitude of the composite effect was not associated with CFMT scores, *r*(200) = .077, *p* = .275 (refer to Figure 4). This was replicated with the residualized CFMT scores, *r*(200) = .064, *p* = .364. When we explored this relationship separately for each participant group, the magnitude of the composite effect was not significantly correlated with CFMT in either Ws (*r*(100) = .089, *p* = .374) or EAs (*r*(98) = .067, *p* = .505) (see Figure 4). The correlation coefficients of both groups were comparable (*z* = .155, *p* = .876). The same result was observed with residualized CFMT scores in Ws (*r*(100) = .088, *p* = .380) and EAs (*r*(98) = .043, *p* = .669), and the correlation coefficients were comparable between participant groups (*z* = .316, *p* = .752).

### Navon's task

Irrespective of cultural groups, participants were faster with global‐congruent (*M* = 479.8 ms, *SD* = 82.4) compared with local congruent (*M* = 501.6 ms, *SD* = 90.2) trials, *t*(201) = −5.592, *p* < .001, *d* = −.393. This pattern mimics the classic Navon's (global precedence) effect reported by previous studies (e.g., Estudillo et al., 2022; Leong et al., 2024; Navon, 1977). The group comparison analyses revealed a comparable GPE in EAs (*M* = .207, *SD* = .418) and Ws (*M =* .124, *SD =* 0.320), *t*(200) = 1.590, *p* = .113, *d* = .224 (see Figure 3). The overall magnitude of the GPE was weakly associated with CFMT scores, *r*(200) = .168, *p* = .017 (refer to Figure 4). This was replicated with the residualized CFMT scores, *r*(200) = .166, *p* = .018. However, when the relationship was explored separately for each participant group, the magnitude of the GPE was correlated with CFMT in Ws (*r*(100) = .239, *p* = .015), but not EAs (*r*(98) = .112, *p* = .267) (see Figure 4). Nonetheless, the correlation coefficients of the GPE and CFMT were comparable between both groups (*z* = .919, *p* = .358). The same result was observed with residualized CFMT scores in Ws (*r*(100) = .239, *p* = .015) and EAs (*r*(98) = .111, *p* = .270), and the correlation coefficients were comparable between participant groups (*z* = .926, *p* = .354).

### Exploratory factor analysis (EFA)

The rule of thumb used to determine a priori sample size for an EFA is a participant to variable ratio of 10:1 (Boutet et al., 2021; Costello & Osborne, 2005), while the same ratio for the current study is 202:12, which equates to approximately 17:1.

A summary of the correlation matrix is given in Table 2. The Kaiser–Meyer–Olkin measure of sampling adequacy (KMO) of the overall scores was .599, above the suggested cut‐off value of .50 (Boutet et al., 2021; Kaiser, 1974). Bartlett's test of sphericity was significant, *χ* ^2^(15) = 73.019, *p* < .001, supporting that our data can be reduced to underlying factors. The exploratory factor analyses identified two main components from the scree plot of our six measures that explained a total variance of 35.2% across all participants (see Table 3). The first component was able to explain a modest portion of the variance (18.1%) and loaded moderately by the scores on the CFMT, the inversion, and part‐whole tasks. The second factor was strongly loaded by CFE, which accounted for 17.1% in variance. In brief, this suggests that there are both overlapping and distinct mechanisms of holistic processing underlying face recognition.

**TABLE 2:** Pearson's correlation analyses between CFMT, CCMT and the four holistic measures of all participants (both EAs and Ws).

Measures	CFMT	CCMT	FIE	PWE	CFE	GPE
CFMT						
CCMT	.130					
FIE	.385***	.114				
PWE	.307***	.081	.193**			
CFE	.077	.066	.012	−.117		
GPE	.168*	.034	.090	.101	−.084	

*Note*: Values represent the coefficient from overall scores (*N* = 202). Coefficients highlighted in grey represent correlations significant at the level of *p < . 05, **p < .01, or ***p < .001.

**TABLE 3:** Results from the principal component analysis (loading matrix and variance explained).

Measures	Factor 1	Factor 2
CFMT	.772	
CCMT		
FIE	.495	
PWE	.409	
CFE		.998
GPE		
Eigenvalues	1.72	1.13
Variance explained (%)	18.1	17.1

*Note*: Only values above the threshold following the rotation are shown.

Abbreviations: CCMT, Cambridge car memory test; CFE, composite face effect; CFMT, Cambridge Face Memory Test; FIE, face inversion effect; GPE, global precedence effect; PWE, part‐whole effect.

## DISCUSSION

The aim of this study was to investigate whether individual differences in face recognition ability can be explained by holistic processing, and whether the underlying cognitive mechanisms of holistic processing are unitary. We found that participants' performances in the inversion, part‐whole, composite, and Navon's tasks replicated the effects commonly reported in the literature (Hole, 1994; Leong et al., 2024; Navon, 1977; Rossion, 2008, 2013; Tanaka & Farah, 1993; Tanaka & Simonyi, 2016; Yin, 1969). In line with previous studies, we also found that the inversion (Belanova et al., 2021; Rezlescu et al., 2017), part‐whole (DeGutis et al., 2013; Rezlescu et al., 2017; Wang et al., 2012), and GPEs (Gerlach & Starrfelt, 2018) were all positively associated with individual differences in FRA, specifically face memory. Importantly, we replicated these patterns after isolating processes specific to face memory by regressing out variance reflecting non‐face object recognition (i.e., CCMT), suggesting that these relationships reflect face‐specific mechanisms.

To our surprise, the GPE showed a weak but significant positive association with FRA, but not with non‐face object recognition (CCMT). Partially supporting Gerlach and Starrfelt (2018), this suggests that domain‐general selective attention plays a small yet meaningful role in predicting individual differences in face memory, but not general visual memory for non‐face objects. This aligns with the notion that faces are *special*, and that perceiving a Gestalt representation does not necessarily enhance recognition for non‐face objects (Farah et al., 1998; McKone & Robbins, 2011). This pattern also partially aligns with Richler, Cheung, et al. (2011), who showed that selective attention, as measured with the complete version of the composite face task, is associated with face recognition. However, the weak correlation between FRA and GPE should be interpreted with caution, as it may be driven purely by the performance of Ws, and the reliability of this effect was also relatively lower in Ws compared with EAs.

In addition, our factor analyses also revealed a shared component between FRA and holistic face processing, independent of individual differences in recognizing non‐face objects (CCMT) and globally processing visual information (GPE). Contrary to other previous findings (Audette et al., 2025; Konar et al., 2010; Richler et al., 2014; Verhallen et al., 2017), our results provide further support for the notion that holistic processing contributes meaningfully to individual differences in FRA. As discussed, the varying correlations (or absence thereof) between holistic processing and FRA across studies may be the result of how holistic processing is measured and/or defined. Notably, we found that the CFE was not correlated with face recognition ability, replicating previous findings (Konar et al., 2010; Verhallen et al., 2017). Furthermore, the CFE also did not load onto the same component as FRA, suggesting that the CFE measures an aspect of holistic processing that does not reflect individual differences in face recognition. Briefly, our findings support the notion that face recognition ability, specifically face memory, can be predicted by holistic processing, though this is dependent on the paradigm used.

In line with previous literature (Rezlescu et al., 2017), we found that the face inversion and part‐whole effects were weakly correlated with each other, while neither was associated with the composite face task. Importantly, our factor analysis revealed that the overall scores on the CFMT, FIE, and PWE loaded onto a single component, which we term as the holistic face processing index: *hf*, while CFE loaded onto a second component. This reveals a specific factor underlying FRA, wherein the CFMT, FIE, and PWE measure an overlapping holistic mechanism that is distinct from CFE. This is consistent with the factor analyses conducted by past research using multiple face processing tasks (Boutet et al., 2021; Verhallen et al., 2017). While we expected the Navon's and composite face tasks to capture the magnitude of domain‐general selective attention (Fitousi, 2015, 2020; Ventura et al., 2021, 2022), our results showed that the GPE was not associated with the CFE. This was inconsistent with the view that the holistic mechanism indexed by the standard CFE primarily reflects domain‐general selective attention, as some past studies would argue. Instead, the standard CFE may index a face‐specific mechanism, even though it does not contribute to individual differences in face memory (see Rossion, 2013). Together, these results reveal that what we traditionally consider holistic processing involves multiple distinct cognitive mechanisms, which contribute differently to face recognition.

The notion that the face inversion, the part‐whole and the composite tasks are tapping into overlapping and/or distinct cognitive mechanisms and may contribute to face recognition ability differently is not surprising, given that there are notable similarity and differences between these tasks (Boutet et al., 2021; DeGutis et al., 2013; Jin et al., 2024; Li et al., 2017, 2019; Rezlescu et al., 2017). For instance, the CFE is based on the magnitude of holistic interference, reflected by the failure to selectively attend to the top half of the face (Jin et al., 2024; Richler & Gauthier, 2014). The PWE is generally demonstrated by the magnitude of facilitation in encoding and/or integration of featural information into a whole (Boutet et al., 2021; Jin et al., 2024; Rezlescu et al., 2017). Conversely, FIE was thought to be an index for the sensitivity towards facial configuration (Leder & Carbon, 2006; Rossion, 2008). For example, recognition of inverted faces was impaired more strongly than upright faces when faces differed in configural relation than when features themselves were different (Leder & Carbon, 2006). Importantly, inverting faces also significantly affects the magnitude of both the part‐whole and composite effects (McKone et al., 2013). This argues that the FIE indexes a general disruption that encompasses multiple holistic mechanisms (Boutet et al., 2021; Gerlach & Mogensen, 2024). Consistent with this, we found that FIE and PWE were indeed measuring overlapping cognitive mechanisms of holistic processing, likely reflecting the integration process of facial features and its configural relation. However, the absence of a relationship between FIE and PWE with CFE may indicate that the holistic interference captured by the standard composite face task does not reflect a perceptual integration mechanism (see below for further discussion). This distinction further supports the growing view that holistic processing is not a *unitary* construct, but rather a set of overlapping and/or distinct mechanisms.

One could argue that observed loading patterns between CFE and FRA could be confounded by the version of CFE that was used here (e.g., Boutet et al., 2021; Richler, Cheung, et al., 2011). In fact, the standard CFE has been shown to measure distinct underlying mechanism than those in the *complete* version of the composite face task (see review by Richler & Gauthier, 2014). However, both Verhallen et al. (2017) and Ventura et al. (2022) showed that holistic processing—indexed with the complete version of the composite face task—was not associated with FRA (but see Richler, Cheung, et al., 2011), nor did it load onto a common factor underlying face processing (i.e., *f*).^3^ In consideration of the current evidence, it is possible that *f* explains the variance from only parts of face processing that are not reflected by the CFE. For instance, all of the face perceptual and/or recognition tasks in Verhallen et al.'s study have a common requirement to integrate facial features across space, while the CFE reflects the inability to suppress the integration process (i.e., interference). In general, the CFE might reflect necessary but yet distinct aspects of holistic processing that are not associated with individual differences in face processing per se (see also Rossion, 2013).

One of the main goals of this study was to understand what are the cognitive mechanisms that these holistic measures underlie, and how they could map onto individual differences in face memory. While we discussed the possible overlapping cognitive mechanisms of the FIE and the PWE, it remains ambiguous for the CFE. Boutet et al. (2021) hypothesized that the standard CFE and FIE may tap into a configural mechanism reflecting the spatial relations between face parts (see also Richler et al., 2012). However, in that study, these two measures failed to load onto a common factor, nor with the configural featural detection task. Accordingly, we also failed to find an association between the PWE and/or FIE with CFE in the current study, suggesting that the standard CFE does not reflect an integrative process of the parts and configural relations (Rossion, 2013). Another view is that the CFE may tap into other cognitive mechanisms that involve general perceptual abilities (e.g., selective attention; Fitousi, 2015, 2020; Ventura et al., 2022). If selective attention is successfully focused on the relevant half of the face, processing of the irrelevant half is reduced, leading to weaker interference and a diminished CFE. In other words, successful selective attention limits the cognitive resources allocated to the irrelevant half, thereby reducing holistic interference (i.e., CFE). For example, Fitousi (2015) found that performance in perceptual judgements of aligned or misaligned composite faces was not affected by irrelevant face parts. Accordingly, Fitousi argued that the attentional mechanisms reflected in the CFE is not unique to faces. However, our findings failed to support this interpretation. Specifically, the GPE, an indicator of domain‐general selective attention, was neither associated with nor loaded onto the same factor as the CFE. This suggests that while selective attention may play a role in the standard CFE, it does not fully account for the cognitive mechanisms underlying the task.

In light of this, what cognitive mechanism could the standard CFE reflect? Answering this question is difficult, given that there have been extensive debates over the last decade on approaches, operational definitions and calculations of CFE and its underlying mechanism (see discussion from Murphy et al., 2017). The current study may not be positioned to directly resolve this debate, but based on our exploratory factor loadings, we propose a *hybrid* interpretation that accommodates key aspects of multiple accounts (e.g., Richler et al., 2012; Rossion, 2013).

Recent studies have argued that the complete CFE reflects an overlearnt attentional (or perceptual) strategy (Richler et al., 2012; Richler, Cheung, et al., 2011; Ventura et al., 2022) that is acquired through extensive experience with faces and reaching adult‐like levels early in development (Cassia et al., 2009; De Heering et al., 2007; Meinhardt‐Injac et al., 2017). Consequently, this aspect of holistic processing may reach a ‘ceiling’ for majority of observers by adulthood, potentially explaining the absence of correlations between the CFE and face memory or other holistic processing measures (Chua & Gauthier, 2020; Gauthier, 2020). While this interpretation has also been used to argue for a domain‐general attentional mechanism (Bukach et al., 2010; Fitousi, 2015, 2020; Gauthier & Tarr, 2002; Ventura et al., 2021, 2022), generalization is less evident in the standard CFE (Jacques & Rossion, 2010; Robbins & McKone, 2007; Schiltz et al., 2010). Given that they are methodologically comparable from the participant's perspective (Jin et al., 2024; Ventura et al., 2019), it is plausible that the complete and standard CFE tap into related mechanisms, with the standard CFE reflecting a more face‐specific *variant* of this attentional process. One possibility is that the CFE indexes an early perceptual‐attentional component of holistic face processing that facilitates efficient face detection/perception, rather than directly supporting face encoding or memory (Hershler & Hochstein, 2005; Taubert et al., 2011; Tsao & Livingstone, 2008). Consistent with this, CFE emerges early in the visual process system (Jacques & Rossion, 2010; Ramon & Rossion, 2012) and relies on low‐spatial frequency information (e.g., stronger CFE for LSF‐filtered faces; Goffaux & Rossion, 2006). From this perspective, the CFE may reflect a necessary component of face expertise, but once acquired, having even ‘more’ of it does not make people better at recognizing faces (Chua & Gauthier, 2020; Ramon & Rossion, 2012; Rossion, 2013; Ventura et al., 2022). In contrast, *hf* may be more closely related to memory‐based integration of facial features and their configurations during encoding and retrieval (see also Ramon & Rossion, 2012). However, we emphasize that these interpretations remain speculative and have not been directly tested.

Accordingly, we believe that another alternative account offers a more compelling explanation for the present findings. A recent principal component analysis by Bobak et al. (2023) found two main components, reflecting response strategies or biases of ‘confirmation’ and ‘elimination’ from different measures of face recognition and perception ability. They found that in these tasks, accuracy from trials in which a target face matched a previously learnt face or a simultaneously presented face loaded on the *confirmation* component. Accuracy from trials in which a target face did not match the learnt or the simultaneously presented face loaded heavily on the *elimination* component. Thus, these two components reflect different cognitive subprocesses of face recognition: telling faces together and telling faces apart, respectively. Importantly, Bobak et al. (2023) found that the CFMT Long Form (CFMT+; Russell et al., 2009), which is akin to our CFMT task, loaded strongly onto the first confirmation component.

Our main component *hf* may reflect similar strategies and/or biases of ‘confirmation’ because target faces were always present in every trial (that also matches the learnt face) of the three tasks that loaded onto *hf* (e.g., CFMT, part‐whole and inversion tasks). In contrast, the standard composite face task used in our study, which consists of non‐matching faces and target‐absent trials, may also load onto the elimination component (Bobak et al., 2023). In the standard composite face task, the irrelevant (bottom) face parts are always different, and the target (top) halves were either same or different. Therefore, half the trials in the composite tasks may load onto the confirmation component, and another half of the trials may load onto the elimination component. In brief, it is possible that our factor loadings are reflecting task demands or response strategies (confirmation in *hf* vs. confirmation/elimination in CFE), rather than differences in underlying cognitive mechanisms. However, this remains speculative because we are unable to examine the trials within each task separately as all the trials in the CFMT, FIE, and PWE were target‐present, but trials in the CFE consisted of both target‐present and absent elements (for discussion, see also Leong et al., 2024). Future studies may include examination of these structures when other variations of these holistic measures with different task constraints are used (e.g., Tree et al., 2025). For instance, in the face inversion and part‐whole tasks, target and test faces can instead be presented sequentially, following the format of the composite face task. If the resulting factor loadings remain consistent with the current study, it would suggest that *hf* reflects a specific holistic face construct underlying FRA, rather than a construct being driven solely by task demands.

The current study is not without limitations. Specifically, our factor analysis was exploratory, and the two components identified only accounted for a small portion of the variance (~35%). Consequently, our mechanistic interpretations should be treated with caution, as our results do not provide definitive evidence for underlying mechanisms. Nevertheless, this modest variance explained was expected, given that we only included one principal measure of face recognition (i.e., CFMT). If face recognition is a product of multiple underlying mechanisms, it would make sense to have only a small portion explained by a common structure shared between different holistic measures and FRA. Additionally, we did not include a measure of other higher‐level cognitive processes, for example, featural processing, in the current factor analysis. Although holistic processing has been argued to be the ‘backbone’ of FRA, recent studies found that featural processing is also important (Audette et al., 2025; Belanova et al., 2021; DeGutis et al., 2013; Dunn et al., 2024; Leong et al., 2023; Tsantani et al., 2020). This would explain why the common factor(s) underlying face processing found across studies have consistently accounted for only a small portion of the variance. Indeed, including the control conditions in our factor analysis accounted for additional variance (~8%) (see Supporting Information S2). Notably, the component *hf* was retained, suggesting that it represents a robust component contributing to face recognition. While our findings indicate that holistic processing is associated with FRA, our findings also support the possibility that other non‐holistic processes also contribute to individual differences in face memory. However, this interpretation should be treated cautiously due to the high within‐task correlations, and the fact that the magnitude of holistic processing (i.e., residuals or effect sizes) was calculated based on performance in these control conditions. In other words, it would make less sense to use performance in one condition as a measure of two distinct processes. Future research could further explore this hypothesis by using more specific measures of featural processing (e.g., aperture paradigms; Murphy & Cook, 2017; Murphy et al., 2020; Leong et al., 2023; or the perceptual integration paradigm; Audette et al., 2025; Gold et al., 2012).

In addition, while the CFMT is one of the most widely used measure of face recognition ability, certain aspects of the test remain ambiguous. For example, it is unclear what the noise stage in the CFMT specifically measures. Duchaine and Nakayama (2006) proposed that noise was added in this stage to force increased reliance on the ‘special mechanisms’ of face recognition, but it is unclear what cognitive mechanism is being reflected here. In fact, this stage was based on McKone et al.'s (2001) study, which demonstrated that adding noise to faces impaired the perception of inverted but not upright faces. Since perceiving inverted faces was thought to rely on featural processing, McKone et al. proposed that adding noise would isolate configural processing. Hence, it is not surprising that the FIE correlates most strongly with the CFMT, as the ‘special mechanism’ participants are forced to rely on during the noise stage and inversion task are similar. Accordingly, the weak to moderate correlations observed between the CFMT and holistic measures might reflect residual links between the mechanisms or strategies employed across tasks (i.e., repeated presentation; Gauthier, 2020), rather than a true representation of shared underlying mechanisms throughout the entire measure. Future studies should further explore the role of the noise stages in the CFMT at mediating the relationship between FRA and holistic processing.

Previous studies have often shown an extremely low reliability within the three gold‐standard measures (Richler et al., 2014, 2015), which limits the detection of meaningful relationships between measures. Specifically, the maximum observable correlation between two variables is constrained by the reliability of those measures (e.g., upper bound of a correlation between two distinct measures is equal to the square root of the product of their respective reliabilities; Nunnally, 1970). In the current study, even if the performances on the CFMT and part‐whole task were perfectly correlated, the maximum observable correlation is ~.59. This underscores the importance of psychometric rigour when interpreting null or modest findings. In light of this, other alternative holistic face measures have been introduced in recent years, such as the Vanderbilt Holistic Processing Test – Face (Richler et al., 2014), the Vanderbilt Part Whole Test (Sunday et al., 2017), or the perceptual integration paradigm (Audette et al., 2025), which have been shown to have better reliability (~.5 to .6) than the traditional holistic measures. Despite that, the observed reliability in these tasks was similar to those found in the current study (.4 to .6). Thus, we argue that the current findings cannot be fully explained by low reliabilities of the holistic measures.

Moreover, the validity of these traditional holistic tasks has been questioned, with some suggesting that high within‐task correlations (i.e., condition of interests and control conditions) indicate that these conditions may reflect the same cognitive processes (Audette et al., 2025). However, we argue that this high within‐task correlations may instead serve as evidence for the two processes tapping into a shared underlying mechanism: the integration of facial features (first‐order information) into a holistic whole (higher order processing). Indeed, successful face learning likely depends on encoding and integrating individual facial features and their configural relations into a holistic representation, which then enhances subsequent recognition of facial features (Leong et al., 2023). Notably, this subsequent featural processing mentioned is shown to be significantly associated with individual differences in FRA. It is therefore not surprising that performance across these conditions within the tasks measuring holistic processing would be associated. Despite showing no association between holistic integration and FRA, Audette et al.'s (2025) results provide support for the idea that learning upright whole faces facilitates later recognition of isolated features, which appears to be modulated by individual differences in face recognition ability. Furthermore, while their definition – which assumes holistic processing primarily reflects integration of internal features – aligns with Rossion's (2013) conceptualization of facial parts as fundamental building blocks of holistic representation, their integration paradigm cannot dissociate true holistic processing from featural processing strategies. Crucially, without disrupting holistic integration (e.g., through feature replacement), their methodology leaves open the possibility that participants merely identified individual features within combined faces.

Lastly, we acknowledge potential concerns about drawing conclusions from results involving two different cultural groups, as cultural differences could potentially explain the small portion of the variance observed in our factor analysis. However, it is important to highlight that including participants from different cultural backgrounds enhances the generalizability of our findings. If holistic processing is fundamental to face recognition and underlies individual differences in FRA, then its influence should be consistent across cultures. Indeed, the reliability of the measures, performance variance, FRA across samples, the magnitude of holistic face processing, and the relationships between FRA and holistic face processing did not differ significantly between the two groups (but see Supporting Information S3 for feature‐specific performance in the part‐whole tasks). This suggests it is unlikely that cultural differences would influence the observed loading patterns. Furthermore, if cultural differences were impacting the factor loadings, we would expect weaker loading patterns. Instead, we observed strong loadings for the CFE. Notably, the tasks that loaded onto the common factors involved only face recognition, suggesting that while non‐face object processing may vary, the mechanisms of holistic face processing are likely to be universal across sample groups. Nonetheless, the primary aim of the current paper is to adopt a more ecological approach to examine the underlying mechanism of holistic face processing, rather than cultural differences. Hence, future research could further examine whether *hf* would potentially differ across cultures.

## CONCLUSIONS

In conclusion, our findings suggest that some aspects of holistic processing can account for individual differences in face recognition ability. Critically, the current study posited that the three traditional measures of holistic processing (e.g., the inversion, part‐whole and composite tasks) are *not unitary* and they are measuring different underlying cognitive mechanisms, as well contribute to face memory differently. Moreover, our results support the existence of multiple mechanisms underlying face recognition, specifically involving distinct measures of holistic processing. In brief, our findings provide possible explanations on why there have been inconsistent findings concerning the relationship between individual differences in face memory and holistic processing. These findings highlight the need to reconsider both the methodological approaches used to assess holistic processing and the theoretical assumption that it reflects a unitary cognitive mechanism.

## AUTHOR CONTRIBUTIONS

**Bryan Qi Zheng Leong:** Conceptualization; investigation; writing – original draft; methodology; validation; visualization; writing – review and editing; software; formal analysis; data curation. **Ahamed Miflah Hussain Ismail:** Supervision; writing – review and editing; writing – original draft; conceptualization; methodology; software; investigation. **Alejandro J. Estudillo:** Conceptualization; investigation; writing – original draft; writing – review and editing; supervision; data curation; software; methodology.

## FUNDING INFORMATION

This research did not receive any specific grant from funding agencies in the public, commercial, or non‐profit sectors.

## CONFLICT OF INTEREST STATEMENT

The authors declare that they have no known competing financial or non‐financial interests, or personal relationships that could have appeared to influence the work reported in this paper.

## Supporting information


**Figure S1.** Raw accuracy and reaction time (Navon's task only) scores of EAs (grey) and Ws (orange).

**Table S1.** Raw accuracy and reaction time (Navon's task only) scores of White (Ws) and Asian (EAs) participants.
**Table S2**. Pearson's correlation analyses between raw accuracy in all tasks, reaction time from Navon's task, and residuals/standardized difference in the four holistic measures of all participants (both EAs and Ws).
**Table S3**. Results from the principal component analysis (loading matrix and variance explained).
**Table S4**. Raw accuracy scores of each feature across the whole and part conditions of the part‐whole task.
**Table S5**. Pearson's correlation analyses between CFMT, residualized CFMT, CCMT and the part‐whole effects for each feature (both EAs and WCs).

## Data Availability

The datasets generated during and/or analysed during the current study are available in the Open Science Framework repository, https://osf.io/mb5ue/?view_only=24b735f17a3e41ec9dfd1562d8e2da0f.

## Footnotes

^1^
While there is an extensive ongoing debate on which of the two versions (standard and complete) of the composite face task is a ‘better’ measure of holistic face processing (Richler, Cheung, et al., 2011; Rossion, 2013; Richler & Gauthier, 2014; see also Murphy et al., 2017), the aim of the current paper is to capture the face‐specific holistic mechanisms as described in Rezlescu et al. (2017). Thus, in this paper, we use composite face effect (CFE) as a general term for the standard version and specified the complete version as complete CFE.

^2^
Throughout this paper, we use *face identification* as a general term encompassing the ability to perceive, match, or recognize facial identity. In contrast, the term *face recognition* (or *face recognition ability*) is used more specifically to refer to memory‐based identification (i.e., face memory), as indexed by tasks such as the CFMT.

^3^
Although additional analyses by Verhallen et al. using *d*‐prime and/or the raw accuracy scores indicated a strong association between CFMT performance with each condition of the complete CFE, supporting the argument that these two tasks tap into a common mechanism (Richler, Cheung, et al., 2011), this association found appears to depend on how the CFE is calculated and does not directly reflect the magnitude of holistic face processing.
