# Supplementary material for: Task‐specific associations between holistic processing and individual differences in face memory
Source: Br J Psychol. 2026 May 13;117(4):1631–56. doi: 10.1111/bjop.70084 (PMC13655931; doi:10.1111/bjop.70084)
Supplement: Supplementary file 1 [file BJOP-117-1631-s002.docx]

**Supplementary Materials**

*S1. Raw data analysis of variance*

We conducted four repeated measures analyses of variance (ANOVA) to test whether there are cultural differences in participants performances across all four holistic measures (see Table S1).

**Table S1.**

*Raw accuracy and reaction time (Navon’s task only) scores of White (Ws) and Asian (EAs) participants.*

| **Tasks/Conditions** | **Ws** | | **EAs** | |
| --- | --- | --- | --- | --- |
|  | *M* | *SD* [95% CI] | *M* | *SD* [95% CI] |
| **CFMT** | 60.46 | 8.33 [58.8, 62.1] | 60.84 | 8.26 [59.2, 62.5] |
| **CCMT** | 45.50 | 8.29 [43.9, 47.1] | 48.46 | 7.07 [47.1, 49.9] |
| **CFMT residuals** | 2.86 × 10^-15^ | 8.32 [-1.63, 1.63] | 2.30 × 10^-16^ | 8.05 [-1.60, 1.60] |
| **Inversion** | | | | |
| Upright | .773 | .118 [.75, .80] | .800 | .107 [.78, .82] |
| Inverted | .523 | .114 [.50, .55] | .550 | .091 [.53, .57] |
| **Part-whole** | | | | |
| Whole | .790 | .097 [.77, .81] | .764 | .093 [.75, .78] |
| Part | .688 | .090 [.67, .71] | .664 | .079 [.65, .68] |
| **Composite** | | | | |
| Same-Aligned | .739 | .153 [.71, .77] | .744 | .157 [.71, .78] |
| Same-Misaligned | .794 | .141 [.77, .82] | .827 | .140 [.80, .86] |
| **Navon (ms)** | | | | |
| Global-Congruent | 491.5 | 87.4 [474.3, 508.7] | 467.9 | 75.6 [452.8, 482.9] |
| Local-Congruent | 513.1 | 96.0 [494.2, 531.9] | 489.9 | 82.7 [473.5, 506.3] |

*Note*. *CFMT* Cambridge face memory test, *CCMT* Cambridge car memory test.

In the inversion task, our ANOVA revealed a significant main effect of conditions, *F*(1, 200) = 883.073, *p* < .001, 𝜂^2^_𝑝_ = .815 (see Figure S1). Participants were better in the upright conditions than the inverted conditions, regardless of cultural groups. There was also a significant main effect of group, *F*(1, 200) = 4.543, *p* = .034, 𝜂^2^_𝑝_ = .022. EAs performed better than Ws in the inversion task, regardless of conditions. Nonetheless, there was no significant interaction effect, *F*(1, 200) = 2.044×10^-4^, *p* = .989, 𝜂^2^_𝑝_ = 1.022×10^-6^. In line with our main analysis, this suggest that both cultural groups have similar magnitudes of inversion effects.

**Figure S1.**

*Raw accuracy and reaction time (Navon’s task only) scores of EAs (grey) and Ws (orange).*

A)

B)

C)

D)

*Note.* The violin plots represent the density distribution of performance across each condition and group for the A) face inversion task, B) part-whole task, C) composite face task, and D) Navon’s task, respectively. The horizontal line within the boxplot represents the mean scores, whilst the top and bottom hinge of the boxplot represent the first and third quartiles. The vertical black line outside of each boxplot represents the 95% confidence interval. Black-filled circles represent the accuracy scores of individual participants that are outside the 95% confidence interval.

In the part-whole task, our ANOVA revealed a significant main effect of conditions, *F*(1, 200) = 300.969, *p* < .001, 𝜂^2^_𝑝_ = .601 (see Figure S1). Participants were better in the whole conditions than the part conditions, regardless of cultural groups. There was also a significant main effect of group, *F*(1, 200) = 4.804, *p* = .030, 𝜂^2^_𝑝_ = .023. Ws performed better than EAs in the part-whole task, regardless of conditions (see Table S1). Nonetheless, there was no significant interaction effect, *F*(1, 200) = .034, *p* = .854, 𝜂^2^_𝑝_ = 1.697×10^-4^. In line with our main analysis, this suggest that both cultural groups had comparable part-whole effects.

In the composite face task, our ANOVA revealed a significant main effect of conditions, *F*(1, 200) = 68.615, *p* < .001, 𝜂^2^_𝑝_ = .225 (see Figure S1). Participants were better in the same-misaligned conditions than the same-aligned conditions, regardless of cultural groups. However, there was no main effect of group, *F*(1, 200) = 1.000, *p* = .318, 𝜂^2^_𝑝_ = .005. Both Ws and EAs were comparable in the composite face task, regardless of conditions (see Table S1). Furthermore, there was also no significant interaction effect, *F*(1, 200) = 2.749, *p* = .099, 𝜂^2^_𝑝_ = .014. In line with our main analysis, this suggests that both cultural groups have similar magnitudes of composite face effects.

Finally, in the Navon’s task, our ANOVA revealed a significant main effect of conditions, *F*(1, 200) = 31.120, *p* < .001, 𝜂^2^_𝑝_ = .135 (see Figure S1). Participants were faster in the global-congruent conditions than the local-congruent conditions, regardless of cultural groups. Furthermore, there were a main effect of group, *F*(1, 200) = 4.192, *p* = .042, 𝜂^2^_𝑝_ = .021. EAs were generally faster than Ws in the Navon’s task, regardless of conditions (see Table S1). Nonetheless, there was also no significant interaction effect, *F*(1, 200) = 0.004, *p* = .948, 𝜂^2^_𝑝_ = 2.150×10^-5^. In line with our main analysis, this suggest that both cultural groups had comparable global precedence effect.

In summary, regardless of conditions, we found that EAs performed better overall than Ws in the inversion task, comparable in the composite task, but worse in part-whole and Navon’s task (see Figure S1). Importantly, we found no significant interaction effects in our ANOVAs. In line with our residual analyses, holistic processing (based on raw data) from the four measures were comparable across both cultural groups. Even though thorough precautions were taken to ensure that the stimuli are matched in distinctiveness, participants completed only own-race face tasks, meaning that any observed differences could still stem from characteristics of the stimuli.

*S2. Exploratory factor analysis for holistic and featural processing*

Given that the two factors in the first EFA only contributed to 35.2% of the variance, this raises questions about the importance of omitted factors such as feature processing. For this reason, we conducted another EFA that included the relevant control conditions to measure featural processing (e.g., part trials in the part-whole task) and how these measures load onto face memory.

A summary of the correlation matrix is shown in Table S2. The KMO of the overall scores was .653, above the suggested cut-off value of .50 (Kaiser, 1974). Bartlett’s test of sphericity was significant, *χ*^2^(45) = 276.061, *p* < .001, supporting that our data can be reduced to underlying factors. The exploratory factor analyses identified four main components from the scree plot of our six measures that explained a total variance of 43.4% across all participants (see Table S3). The first component was able to explain a modest portion of the variance (12.4%) and loaded moderately by the scores on the CFMT, FIE, PWE, and misaligned accuracies. The second factor was strongly loaded by part accuracy, which accounted for 11.8% in variance. The third factor was strongly loaded by the inverted accuracy, which accounted for 11.1% in variance. Lastly, the fourth factor was loaded modestly by the GPE and strongly by the speed in local-congruent trials, accounting for the remaining 8.1%.

**Table S2.**

*Pearson’s correlation analyses between raw accuracy in all tasks, reaction time from Navon’s task, and residuals/standardized difference in the four holistic measures of all participants (both EAs and Ws).*

| **Measures** | **CFMT** | **CCMT** | **FIE** | **Inverted** | **PWE** | **Part** | **CFE** | **Same-misaligned** | **GPE** | **Local-congruent** |
| --- | --- | --- | --- | --- | --- | --- | --- | --- | --- | --- |
| **CFMT** |  |  |  |  |  |  |  |  |  |  |
| **CCMT** | .130 |  |  |  |  |  |  |  |  |  |
| **FIE** | .385*** | .114 |  |  |  |  |  |  |  |  |
| **Inverted** | .286*** | .153* | .000 |  |  |  |  |  |  |  |
| **PWE** | .307*** | .081 | .193** | .170* |  |  |  |  |  |  |
| **Part** | .453*** | .207** | .434*** | .283** | .000 |  |  |  |  |  |
| **CFE** | .077 | .066 | .012 | .095 | -.117 | .178* |  |  |  |  |
| **Same-misaligned** | .410*** | .143* | .267*** | .310*** | .179* | .244*** | .000 |  |  |  |
| **GPE** | .168* | .034 | .090 | .091 | .101 | .116 | -.084 | .045 |  |  |
| **Local-congruent** | -.047 | .022 | .058 | -.061 | .053 | -.042 | -.152* | -.095 | .308*** |  |

*Note.* Values represent the coefficient from overall scores (*N* = 202). Coefficient scores with .000 represent a value smaller than .001. Coefficients highlighted in grey represent correlations significant at the level of **p* <. 05, ***p* <.01, or ****p< .*001.

**Table S3.**

*Results from the principal component analysis (loading matrix and variance explained).*

| **Measures** | **Factor 1** | **Factor 2** | **Factor 3** | **Factor 4** |
| --- | --- | --- | --- | --- |
| CFMT | .625 |  |  |  |
| CCMT |  |  |  |  |
| FIE | .452 |  |  |  |
| Inverted accuracy |  |  | .990 |  |
| PWE | .554 |  |  |  |
| Part accuracy |  | .907 |  |  |
| CFE |  |  |  |  |
| Same-misaligned accuracy | .461 |  |  |  |
| GPE |  |  |  | .415 |
| Local-congruent RT |  |  |  | .757 |
| **Eigenvalues** | **2.502** | **1.446** | **1.119** | **1.033** |
| ***Variance explained (%)*** | ***12.4*** | ***11.8*** | ***11.1*** | ***8.1*** |

*Note.* Only values above the threshold following the rotation are shown. *CFMT* Cambridge face memory test, *CCMT* Cambridge car memory test, *FIE* face inversion effect, *PWE* part-whole effect, *CFE* composite face effect, *GPE* global precedence effect

As expected, all the control conditions in our holistic face tasks were associated with each other, revealing that these conditions were likely to reflect the same process – featural processing. Furthermore, all the control conditions in our holistic face tasks were also correlated with face and non-face memory. First, the correlations show that non-holistic face processing predicts individual differences in face memory, suggesting the importance of featural processing in face recognition (Leong et al., 2023; DeGutis et al., 2013). Second, the associations between control conditions and CCMT aligns with the notion that non-face objects are processed more featurally (Robbins & McKone, 2007). However, these associations were only weak at best (*r* ≤ .2). Surprisingly, we found no association between CCMT and Local-congruent performance. This argues that general global processing may not contribute to non-face object memory.

Drawing similarity with our main EFA, where CFMT, PWE, and FIE loaded onto the first factor (i.e., *hf*). Here, the misaligned accuracy also loaded onto *hf*. In contrast, the second CFE factor disappeared. Surprisingly, our factor analyses revealed that not all the control conditions in face tasks loaded onto each other. Despite that our correlational analyses revealed that all the control conditions in these holistic face measures are correlated, as well as with face memory, they are not of the same underlying construct. As expected, the GPE and local performance loaded onto a fourth factor, likely to represent a general-domain global processing component. In brief, the EFA here suggest that featural processing, but not non-face object recognition, contributes also to face recognition and was able to explain additional variance beyond holistic processing. Importantly, similar to holistic processing, there was also multiple distinct face-specific mechanisms underlying featural processing.

We propose two explanations here for loading patterns observed for face-specific tasks: 1) the loading pattern may reflect the number of facial features available for identification. For instance, in the ‘part’ trials, only one feature of any one identity was shown. Thus, participants could be focusing on finer details within each feature, e.g., lip wrinkles etc. In contrast, the CFMT, FIE, PWE, and misaligned present full upright faces, enabling participants to process multiple features (and likely their configurations). Although inverted faces also contain multiple features, inversion likely disrupts processing of individual features as well (Gerlach & Morgensen, 2024).

Alternatively, 2) this pattern could be the result of the specific features used to *facilitate* face identification. In the misaligned condition (which loaded onto the *hf*), participants were required to only identify the top half of the face (i.e., eyes) without interference from the bottom-irrelevant half. In this sense, the misalignment is actually facilitating identification. In addition, it is well known that the eye region is the most diagnostic region of the face (Ellis et al., 1979; Rossion, 2013). Participants’ performance in the CFMT, PWE, FIE, and misaligned accuracy can be purely driven by identification of the eye region, which is not possible in the part and inverted trials. For example, the part and inverted conditions most likely require identification of all diagnostic features equally, e.g., eyes, nose, mouth. Consequently, the CFE did not load onto any of these components because it measures interference towards identification, rather than facilitation. Where observed, however, we recommend that these interpretations should be treated with caution.

*S3. Feature-specific analyses in the part-whole task*

To examine if the two groups differ in their identification accuracy for each feature (eyes, nose, mouth) and conditions (whole, part) separately in the part-whole task, a mixed-factorial ANOVA was conducted on the raw accuracy scores. We found a significant main effect of condition (*F*(1, 200) = 300.969, *p* < .001). Regardless of cultural groups, participants were better at recognizing whole faces compared to individual parts (*t*(201) = 17.348, mean difference = .101). We also found a significant main effect of features (*F*(2, 400) = 246.418, *p* < .001), where participants were best at recognizing the eyes, followed by the mouth, and nose, regardless of cultural groups and condition. Specifically, participants were more accurate in the eyes condition than the mouth (*t*(201) = 17.272, *p* < .001, mean difference = .120) or the nose conditions (*t*(201) = 20.358, *p* < .001, mean difference = .157), and more accurate in the mouth than nose conditions (*t*(201) = 4.879, *p* < .001, mean difference = .037). There was also a significant group effect, where Ws participants were better than EAs in the part-whole task (*F*(1, 200) = 4.804, *p* = .030; mean difference = .025). We also found a significant interaction effect between features and groups (*F*(2, 400) = 5.710, *p* = .004). Post-hoc tests with Holm-Bonferroni corrections revealed that Ws were better at recognizing the nose conditions (*p* = .002), but comparable for the eyes and mouth, regardless of conditions. A second interaction effect was found for conditions and features, *F*(2, 400) = 5.300, *p* = .005), wherein participants (regardless of race) were better at recognizing the eyes than any other features in both whole and part conditions, and were also better for the mouth than nose in the whole conditions, but not the part conditions (see Table S4). Nonetheless, we did not find a three-way interaction (*F*(2, 400) = .397, *p* = .673), suggesting that both groups have comparable accuracies in each conditions × features, as well as overall part-whole effect.

**Table S4.**

*Raw accuracy scores of each feature across the whole and part conditions of the part-whole task.*

| **Measures** | **Whole** | | | **Part** | | |
| --- | --- | --- | --- | --- | --- | --- |
|  | **Eyes-Nose** | **Eyes-Mouth** | **Nose-Mouth** | **Eyes-Nose** | **Eyes-Mouth** | **Nose-Mouth** |
| Mean difference | .160*** | .105*** | -.055*** | .154*** | .136*** | -.018 |
| *t* | 16.141 | 12.204 | -5.498 | 15.860 | 14.132 | 1.937 |

*Note.* Mean differences highlighted in grey represent differences significant at the level of **p* <. 05, ***p* <.01, or ****p< .*001 (with Holm-Bonferroni corrections).

To further examine if there are cultural differences in the holistic processing for specific features, we also compared the residuals between Ws and EAs for each feature (DeGutis et al., 2012; Leong et al., 2024). Our analyses revealed no differences in part-whole effect between Ws and EAs for the eye (Ws: *M =* .005, *SD =* .083, EAs: *M =* -.005, *SD =* .087), *U* = 5373.500, *p* = .511, *r*_rb_ = .054, 95% CI *r*_rb_ = [-.106, .210]) and mouth (Ws: *M =* .001, *SD =* .109, EAs: *M =* -.001, *SD =* .129), *U* = 5029.000, *p* = .865, *r*_rb_ = -.041, 95% CI *r*_rb_ = [-.172, .145]) regions. ^[1]^ However, we found that Ws had significantly larger part-whole effects for the nose (*M =* .017, *SD =* .132) compared to EAs (*M =* -.017, *SD =* .121), *U* = 5029.00, *p* = .023, *r*_rb_ = .185, 95% CI *r*_rb_ = [.028, .334]).

To account for this cultural distinction in feature processing, we also correlated residuals for each feature (separated by cultural groups) against the CFMT, residualised CFMT, CCMT scores (face and non-face memory, respectively), using Pearson’s tests. (see Table S5). Overall, face memory (as reflected by CFMT and residualised CFMT) was generally associated with the part-whole effect for the eyes, nose, and mouth in Ws, but only eyes and mouth in EAs. Furthermore, non-face object memory was generally not correlated with facial features in both cultural groups, except the nose region in Ws.

**Table S5.**

*Pearson’s correlation analyses between CFMT, residualised CFMT, CCMT and the part-whole effects for each feature (both EAs and WCs).*

| **Measures** | **WCs** | | | **EAs** | | |
| --- | --- | --- | --- | --- | --- | --- |
|  | **Eyes** | **Nose** | **Mouth** | **Eyes** | **Nose** | **Mouth** |
| **CFMT** | | | | | | |
| *r* | .276** | .338*** | .302** | .323** | .161 | .288** |
| Fisher’s *z* | .283 | .352 | .312 | .335 | .163 | .297 |
| **CCMT** | | | | | | |
| *r* | .157 | .293** | .135 | .081 | -.110 | -.034 |
| Fisher’s *z* | .159 | .301 | .136 | .081 | -.111 | -.034 |
| **CFMT residual** | | | | | | |
| *r* | .268** | .324*** | .295** | .313** | .191 | .303** |
| Fisher’s *z* | .275 | .336 | .305 | .324 | .193 | .313 |

*Note.* Values represent the coefficient from overall scores (*N* = 102 and 100 for WCs and EAs, respectively). Coefficients highlighted in grey represent correlations significant at the level of **p* <. 05, ***p* <.01, or ****p< .*001.

Together, our feature-specific analysis suggests that there are subtle cultural differences in holistic processing, particularly for specific facial features, e.g., the nose region. Nonetheless, the difference here could also be the result of non-face object recognition ability (as reflected by significant correlation of the nose-residuals and CCMT in Ws but not EAs). Overall, these findings are limited to own-race faces and could be the result of other non-identity components, future research should further examine if the mixed findings in literature can be explained by feature-specific holistic processing by following a similar approach here.

# References

1. Kaiser, H. F. (1974). An index of factorial simplicity. *Psychometrika, 39*(1), 31–36.
2. Leong, B. Q. Z., Estudillo, A. J., & Hussain Ismail, A. M. (2023). Holistic and featural processing’s link to face recognition varies by individual and task. *Scientific Reports*, *13*(1), 16869.
3. DeGutis, J., Wilmer, J., Mercado, R. J., & Cohan, S. (2013). Using regression to measure holistic face processing reveals a strong link with face recognition ability. *Cognition*, *126*(1), 87-100.
4. Robbins, R., & McKone, E. (2007). No face-like processing for objects-of-expertise in three behavioural tasks. *Cognition*, *103*(1), 34-79.
5. Ellis, H. D., Shepherd, J. W., & Davies, G. M. (1979). Identification of familiar and unfamiliar faces from internal and external features: Some implications for theories of face recognition. *Perception*, *8*(4), 431-439.
6. Rossion, B. (2013). The composite face illusion: A whole window into our understanding of holistic face perception. *Visual Cognition*, *21*(2), 139-253.
7. DeGutis, J., Cohan, S., Mercado, R. J., Wilmer, J., & Nakayama, K. (2012). Holistic processing of the mouth but not the eyes in developmental prosopagnosia. *Cognitive Neuropsychology*, *29*(5-6), 419-446.
8. Leong, B. Q. Z., Hussain Ismail, A. M., Wong, H. K., & Estudillo, A. J. (2024). The heterogeneity of holistic processing profiles in developmental prosopagnosia: holistic processing is impaired but not absent. *Cognitive Neuropsychology*, *41*(3-4), 129-147.

1. While the assumption for equal variances was met using Levene’s test (*ps* > .05), the test of normality using Shapiro-Wilk test were found to be significant in the part-whole effect for the three features (*ps* < .05). For this reason, a Mann-Whitney U *t*-test was conducted instead to compare the residual scores for each feature between the cultural groups.
